# Analysis of Hemorrhagic Fever With Renal Syndrome Using Wavelet Tools in Mainland China, 2004–2019

**DOI:** 10.3389/fpubh.2020.571984

**Published:** 2020-12-01

**Authors:** Lu-Xi Zou, Ling Sun

**Affiliations:** ^1^School of Management, Zhejiang University, Hangzhou, China; ^2^Department of Nephrology, Xuzhou Central Hospital, The Xuzhou School of Clinical Medicine of Nanjing Medical University, Xuzhou, China; ^3^Xuzhou Clinical School of Xuzhou Medical University, Xuzhou, China

**Keywords:** hemorrhagic fever with renal syndrome, geographical information system, wavelet power spectrum, wavelet neural network, public health

## Abstract

**Introduction :** Hemorrhagic fever with renal syndrome (HFRS) is a life-threatening public health problem in China, accounting for ~90% of HFRS cases reported globally. Accurate analysis and prediction of the HFRS epidemic could help to establish effective preventive measures.

**Materials and Methods :** In this study, the geographical information system (GIS) explored the spatiotemporal features of HFRS, the wavelet power spectrum (WPS) unfolded the cyclical fluctuation of HFRS, and the wavelet neural network (WNN) model predicted the trends of HFRS outbreaks in mainland China.

**Results :** A total of 209,209 HFRS cases were reported in mainland China from 2004 to 2019, with the annual incidence ranged from 0 to 13.05 per 100,0000 persons at the province level. The WPS proved that the periodicity of HFRS could be half a year, 1 year, and roughly 7-year at different time intervals. The WNN structure of 12-6-1 was set up as the fittest forecasting model for the HFRS epidemic.

**Conclusions :** This study provided several potential support tools for the control and risk-management of HFRS in China.

## Introduction

Hemorrhagic fever with renal syndrome (HFRS) is a rodent-borne infectious disease caused by hantaviruses in Europe and Asia (1, 2). China has the highest incidence of HFRS and reported ~90% of HFRS cases globally in the last few decades (3). Preventive and management measures have been implemented and played essential roles in HFRS control, including rodent elimination, vaccination to a high-risk population, and health education (1, 4). While there were a total of 209,209 HFRS cases and 1,855 deaths reported during 2004–2019. Epidemiological surveillance of the temporal and spatial distribution of HFRS contributes to identifying its outbreak regularity, epidemic areas, and high-risk populations. Therefore, statistical models are needed to describe and forecast HFRS outbreaks accurately, which is essential for reducing HFRS incidence.

Several studies have been performed to establish models to analyze the HFRS epidemic in several provinces of China (5–7). This study aimed to investigate the current epidemic situation of HFRS in mainland China using multi-dimensional methods. The geographical distribution of HFRS was analyzed using geographic information system (GIS), the periodicity of HFRS was detected using wavelet power spectrum (WPS), and the trend of HFRS outbreaks was forecasted by wavelet neural network (WNN). The WPS and WNN are applied to analyze and predict the HFRS outbreaks in mainland China for the first time. These findings could be helpful to optimize preventive interventions against the HFRS epidemic.

## Materials and Methods

### Data Sources and Collection

The national data on monthly HFRS cases were obtained from the Chinese Center for Disease Control and Prevention (CCDC), from January 2004 to December 2019 (www.chinacdc.cn). The province-level data (from 2004 to 2018) on annual HFRS cases were from the China Public Health Statistical Yearbook, from 2005 to 2019. There was no HFRS data from Macao, Taiwan, and Hong Kong. In mainland China, HFRS is a notifiable infectious disease of class B, and all confirmed HFRS cases should be reported to CCDC in 48 h through the National Notifiable Infectious Diseases Reporting Information System (8).

### Geographical Information System (GIS) Mapping

To analyze the spatiotemporal distribution of the HFRS epidemic, a map of China at provincial administrative regions and 1:1,000,000 scale was from the National Natural Resources and Geospatial Basic Information Database (www.geodata.gov.cn). According to the average annual incidence of HFRS, all provinces were divided into five categories: very high endemic areas with incidences >5.0/100,000 persons; high endemic areas with incidences between 1.0 and 5.0/100,000 persons; medium endemic areas with incidences between 0.5 and 1.0/100,000 persons; low endemic areas with incidences between 0.01 and 0.5/100,000 persons; very low endemic areas with incidences between 0 and 0.01/100,000 persons, and No data areas; respectively. On the maps, these five types of categories were color-coded. The annual province-level HFRS incidence was analyzed separately and then mapped using ArcGIS software (version 10.4, ESRI Inc., Redlands, CA, USA).

### Wavelet-Based Function Approximation

The wavelet-based function is the foundation of the WPS and WNN analysis (9). The principle of wavelet transform is that the signals at different time scales could be decomposed into a set of wavelet-based functions, and this set ψ_*a,b*_ can be generated by translating and scaling the mother wavelet function ψ, according to:

(1) $$\begin{array}{l} \psi_{a,b}=|a{|}^{-\frac{1}{2}}\psi \left(\frac{x-b}{a}\right),\left(a,b\epsilon R,a\neq 0\right) \end{array}$$

In which ψ_*a,b*_ is called analysis wavelet or continuous wavelet; where *a* is the scale parameter which adjusts the dilation of the wavelet, and *b* determines the location of the wavelet (10).

ψ_*a,b*_ is a family of functions generated from one single function ψ(*x*) by the operation of dilation and translation, ψ (*x*) ∈ *L*^2^(R) is called a mother wavelet function that satisfies the admissibility condition:

(2) $$\begin{array}{l} C_{\psi }\;=\int_{0}^{+\infty }\frac{{\left|\hat{\psi }\left(\omega \right)\right|}^{2}}{\omega }d\omega <+\infty \end{array}$$

where $\hat{\psi }\left(\omega \right)$ is the Fourier transform of ψ(*x*).

Grossmann and Morlet (11) proved that any function (*x*) in *L*^2^(R) can be represented by:

(3) $$\begin{array}{l} f\left(x\right)\;=\frac{1}{C_{\psi }}{\iint Wf\left(a,b\right)|a|}^{-\frac{1}{2}}\psi \left(\frac{x-b}{a}\right)\frac{1}{a^{2}}dadb \end{array}$$

Where (*a, b*) given by:

(4) $$\begin{array}{l} Wf\left(a,\;b\right)\;=|a{|}^{-\frac{1}{2}}\int_{-\infty }^{+\infty }\psi \left(\frac{x-b}{a}\right)f\left(x\right)dx \end{array}$$

Where *Wf*(*a, b*) is the continuous wavelet transform of *f* (*x*) (12).

### Wavelet Power Spectrum

In analogy with the terminology used in the Fourier case (9), the (local) WPS (sometimes called *scalogram* or *wavelet periodogram*) is defined as:

(5) $$\begin{array}{l} {\left(WPS\right)}_{f}\left(a,\;b\right)\;=|w_{f}\left(a,\;b\right){|}^{2} \end{array}$$

The WPS may be averaged over time for comparison with classical spectral methods. When the average is taken over all times, the *global wavelet power spectrum (GWPS)* is obtained from:

(6) $$\begin{array}{l} {\left(GWPS\right)}_{f}\left(b\right)\;=\int_{-\infty }^{\infty }|w_{f}\left(a,\;b\right){|}^{2}da \end{array}$$

### Wavelet Neural Network

**Figure 1:**
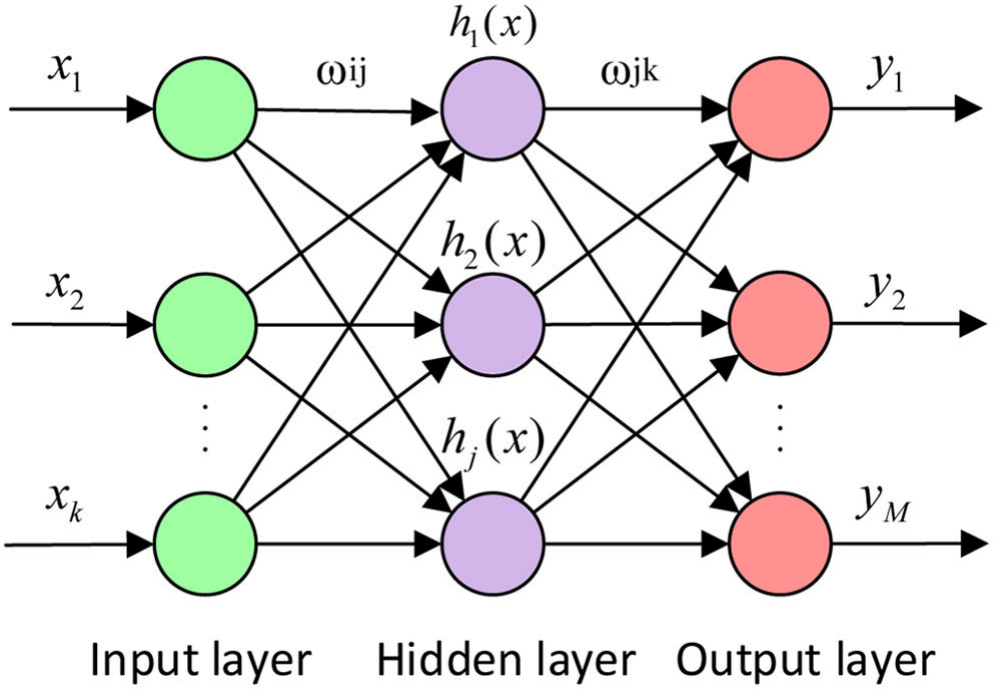
The topological structure of wavelet neural network.

The artificial neural network (ANN) is a computing system made up of several simple and highly interconnected processing elements, those mimic neurons and process information through their dynamic state responses to external input (13). ANN is equivalent to network operation, that consists of different layers, an input layer, a hidden layer (black box), and an output layer (Figure 1). WNN is based on the topology of the back-propagation (BP) neural network, the wavelet-based functions are considered as the transfer functions of the hidden layer nodes, and its learning process consists of two processes: forward propagation and reverses propagation (14, 15).

Figure 1 shows the topological structure of the WNN. X_1_, X_2_, …, X_k_ is the input parameters of WNN, Y_1_, Y_2_, …, Y_m_ is the prediction output of the WNN, and the ω_ij_ and the ω_jk_ are the weights of the WNN. The input signal sequence is X_i_ (i = 1, 2, …, K), the formula for calculating the output of the hidden layer is:

(7) $$\begin{array}{l} h\left(j\right)=hj\left(\frac{\sum_{i=1}^{k}\omega_{ij}-b_{j}}{a_{j}}\right)\;j=1,2,\cdots l \end{array}$$

Of which, *h(j)* is the output value of *j* in the hidden layer; ω_ij_ is the connection weights of the input layer and the hidden layer; a_j_ is the expansion factor of the wavelet base function h_j_; b_j_ is the translation factor of h_j_; h_j_ is the wavelet basis function; *l* is the number of hidden layer nodes. In this study, we used the Morlet mother wavelet function as the wavelet base function (16). Its mathematical formula is:

(8) $$\begin{array}{l} y=\cos\left(1.75x\right)e^{-\frac{x^{2}}{2}} \end{array}$$

**Figure 2:**
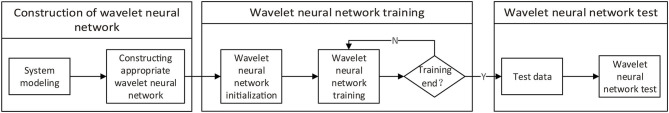
The algorithm flow chart of wavelet neural network.

Figure 2 shows the algorithm flow chart of the WNN. All analyses are conducted using MATLAB (v. 2016, MathWorks, Inc., USA).

To evaluate the accuracy of the algorithm of WNN in predicting HFRS incidence, the mean squared error (MSE), relative mean squared error (RMSE), normalized root mean squared error (NRMSE), and mean absolute percentage error (MAPE) are calculated, whose formulae are as follows (17):

(9) $$\begin{array}{l} MSE=\frac{1}{n}\overset{n}{\underset{t=1}{\sum }}|actual\left(t\right)-forecast\left(t\right)| \end{array}$$

(10) $$\begin{array}{l} RMSE=\sqrt{\frac{{\sum_{t=1}^{n}|actual\left(t\right)-forecast\left(t\right)|}^{2}}{n}} \end{array}$$

(11) $$\begin{array}{l} NRMSE=\frac{RMSE}{forecast\left(\max\right)-forecast\left(\min\right)} \end{array}$$

(12) $$\begin{array}{l} MAPE=\frac{1}{n}\overset{n}{\underset{t=1}{\sum }}\left|\frac{actual\left(t\right)-forecast\left(t\right)}{actual\left(t\right)}\right|\times 100\% \end{array}$$

Where *actual*(*t*) denotes the *t*th actual value, and *forecast*(*t*) denotes the *t*th predicted value. The *forecast*(max) *and forecast*(min) denote the *maximum and minimum* predicted value respectively.

## Results

### Spatiotemporal Distribution of HFRS Outbreak in Mainland China, 2004–2018

**Figure 3:**
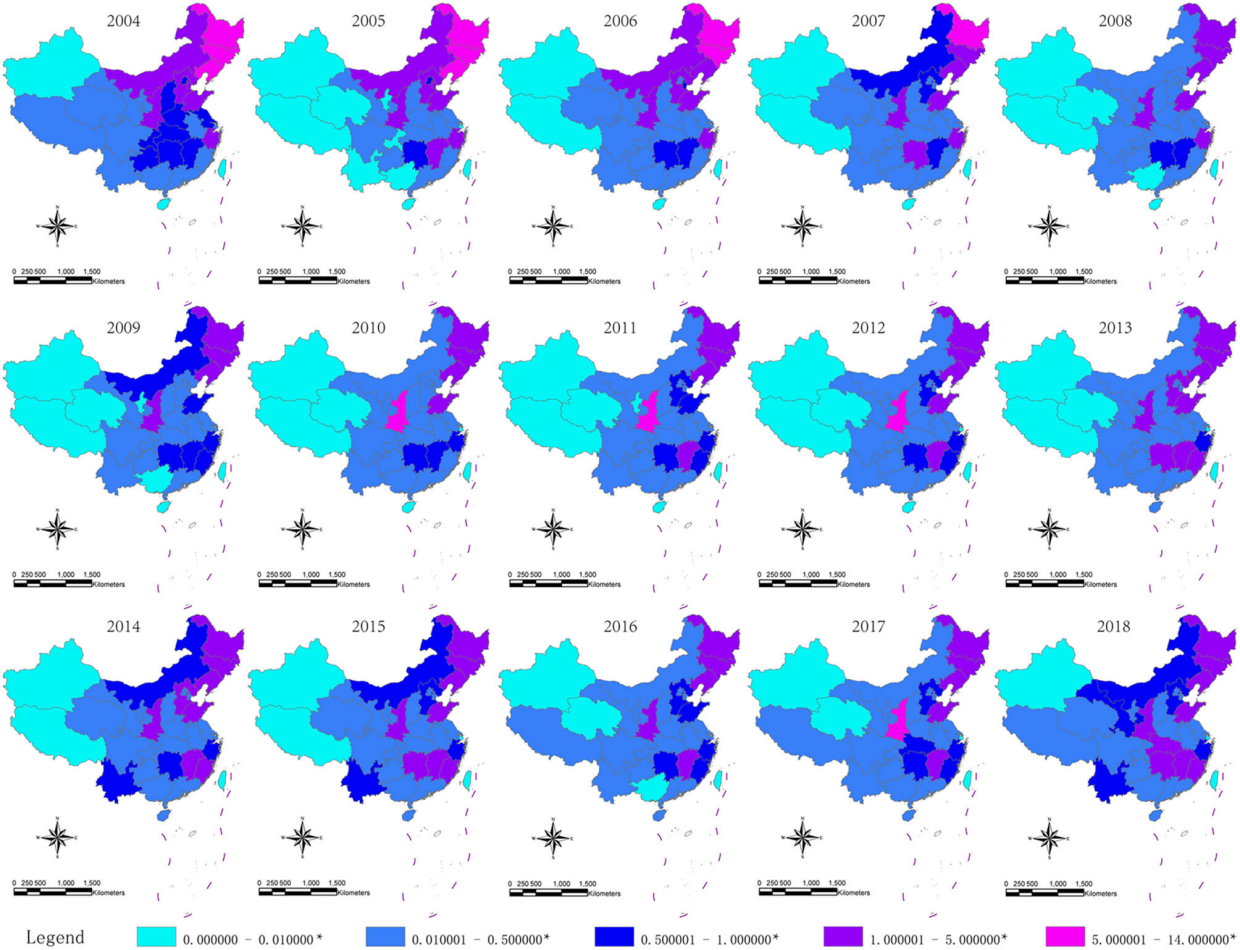
Yearly distribution of HFRS incidence in China, 2004–2018. *Per 100,000 individuals. Geographical information system (GIS) maps are generated by software ArcGIS using the data of China Public Health Statistical Yearbook.

A total of 199,092 HFRS cases were reported from 2004 to 2018 in mainland China. The annual province-level incidence of HFRS ranged from 0 to 13.05 per 100,000 persons. Figure 3 showed that Xinjiang and Tibet had the lowest incidence. Xinjiang was non-endemic except for 2009 and 2017, with HFRS incidence of 0.01/100,000 persons in both years. Tibet was non-endemic from 2005 to 2015, while its HFRS incidence was 0.04/100,000 persons in 2004, and 0.03/100,000 persons in 2016, 2017, and 2018. In 2004, Jilin, Heilongjiang, and Liaoning had the highest HFRS incidence, and Hebei, Inner Mongolia, Jiangxi, Shandong, Shaanxi, and Zhejiang ranked second place. Since 2005, the HFRS incidence had declined in these provinces except for Shaanxi. In 2010, 2011, 2012, 2017, and 2018, Shaanxi surpassed all traditional epidemic areas and ranked first place with the highest incidence.

### WPS Analysis

**Figure 4:**
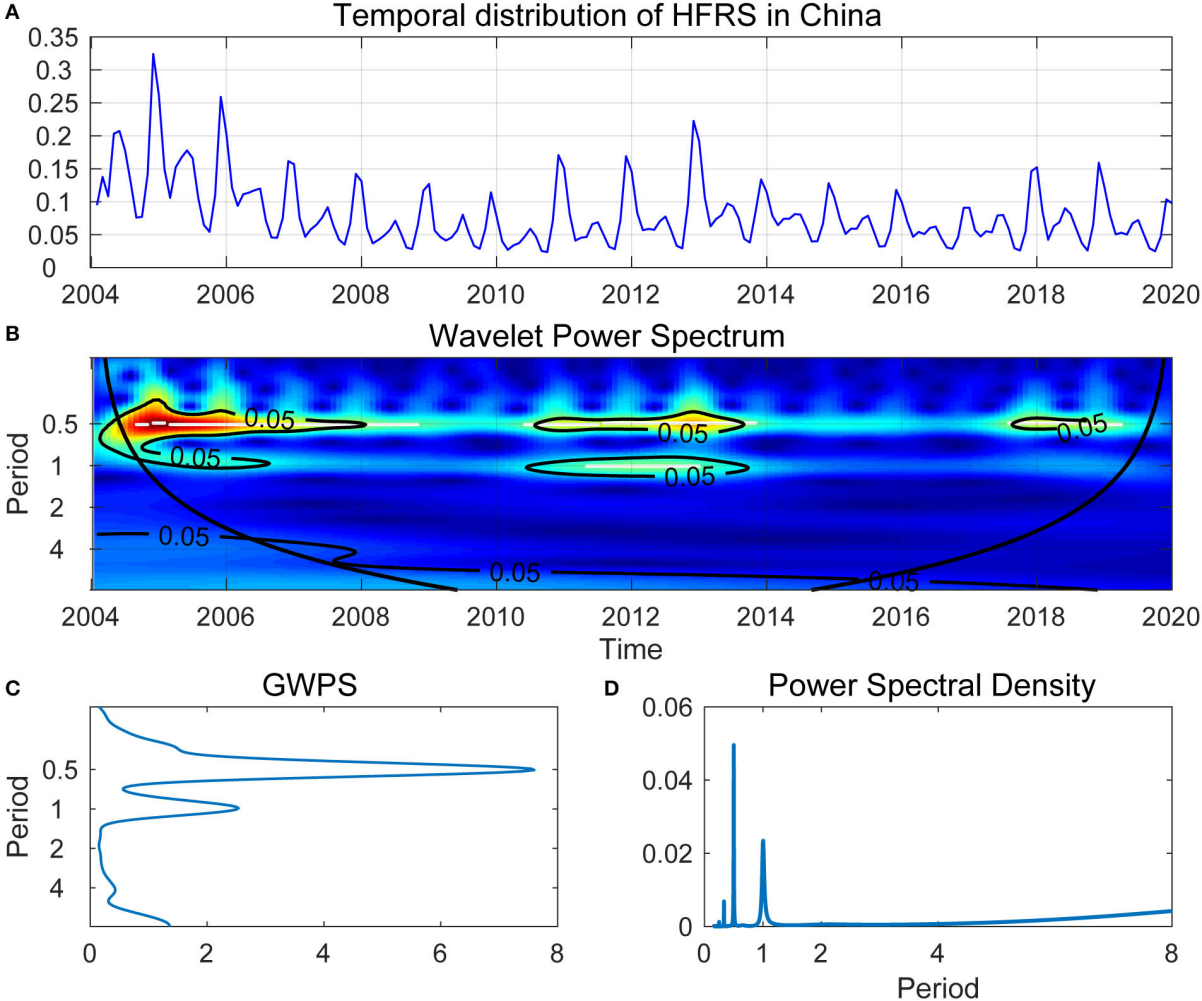
**(A)** Temporal distribution of HFRS in China. **(B)** Wavelet Power Spectrum (WPS) of the reported monthly HFRS outbreaks. The cone of influence was separated by thick black lines, indicating the regions affected by edge effects. The color code for power ranged from blue (low power) to red (high power). The white lines showed the maxima of undulations in the WPS. **(C)** Global wavelet power spectrum (GWPS) of HFRS outbreak—average wavelet power for each frequency. **(D)** Fourier power spectral density of HFRS outbreaks, the peaks of power around half a year and 1 year can be observed in **(C,D)**.

Analyses of the WPS unfolded the cyclical fluctuation of the HFRS epidemic in mainland China. Figure 4A showed the time series of monthly HFRS incidence in mainland China between January 2004 and December 2019. Figure 4B showed the WPS of monthly HFRS incidence illustrated in Figure 4A. In Figure 4B, the horizontal axis presented the time dimension, and the vertical axis presented the period, which was the outbreak cycles of HFRS. The color gave power. Blue to red color-coding indicated increasing power, and warm colors represented high power. Between 2004 and 2019, the time series of annual HFRS incidence in mainland China generated a peak in power around 0.5, indicated that the cyclical fluctuation of HFRS epidemic was a half year during 2004–2008, 2011–2013, and 2018. The 1-year period cycle was observed in the years 2004–2006 and 2011–2013. Between the year 2004 and 2019, there was also a light color region close to 7–8, indicated a 7- or 8-year cyclical fluctuation of the HFRS epidemic during this period.

Furthermore, for ease of interpretation, we converted frequencies into period cycles. GWPS (Figure 4C) presented the same information as the WPS (Figure 4D). However, Figure 4D showed that the information on the transient dynamics could be missed, the estimate of power spectral density captured both the 0.5- and the 1-year cycles but failed to capture the 7- or 8-year cycle when using the traditional spectral analysis.

### WNN for HFRS Forecasting

**Figure 5:**
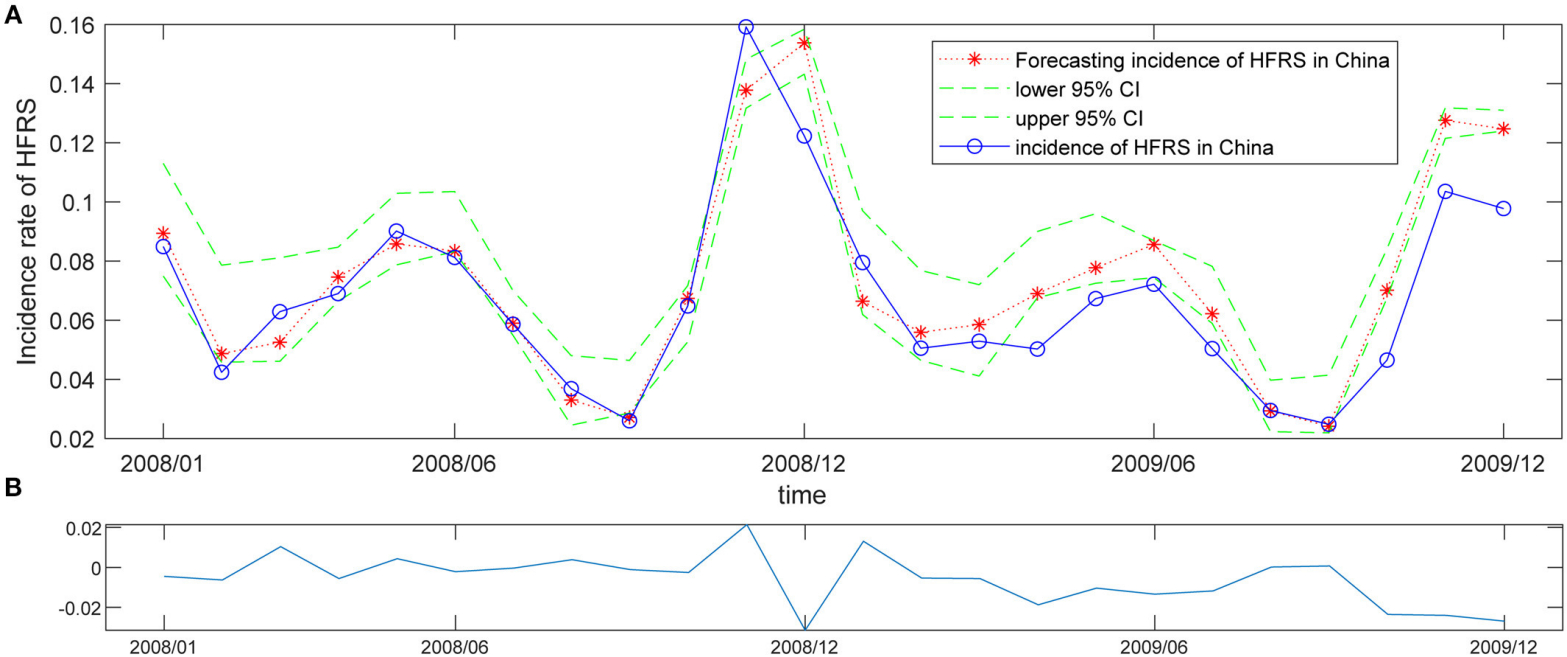
Prediction of HFRS incidence with the WNN model of 12-6-1. **(A)** Forecasted value of HFRS incidence according to the WNN model of 12-6-1. The red asterisk and dashed line represent the forecasted counts, the blue solid line represents the observed values, and the green dashed lines above and below the red dashed line from Jan 2008 to Dec 2019 indicate the upper and lower 95% confidence limits (CL), respectively, for the forecasted counts. **(B)** The absolute error of this WNN model in the training process.

The ANN is not only a mathematical model but also a supervised learning algorithm, that distributes parallel information processing based on the behavior characteristics of animal neural network. WNN is one kind of ANN consists of many simple connected processors called neurons or units, and linked by directed connections. The dataset of monthly HFRS incidence was split into a training period (2004/1–2017/12), and a validation period (2018/1–2019/12), the latter was used to test the predictive ability of the WNN model. First, we initialized the weights of network and wavelet function parameters, then set the weight function-learning rate to 0.1, the wavelet-based parameters learning rate to 0.01, the maximum error to 0.001. To compare the accuracy of WNN models with different structures, we calculated the MSE, RMSE, NRMSE, and MAPE for each WNN structure, then select the fittest model with a minimum value of MSE (RMSE, NRMSE, or MAPE) (Supplementary Table 1). Finally, the WNN structure was determined to be 12-6-1, that was, the input layer had 12 nodes, which meant that the incidence of 12 months before the set time point, hidden layer had 6 nodes, and the output layer has 1 node. Network training was repeated 1,000 times. Figure 5A showed the trends of WNN forecasting values and actual observed values. The WNN was used to train 1,000 times with the data, and the absolute error of the WNN in the training process was shown in Figure 5B. The relative error for HFRS fluctuation from this WNN model was narrow, indicated that the introduction of wavelet function could improve the prediction efficiency of the neural network.

## Discussion

HFRS is an infectious disease caused by hantaviruses, with relatively high prevalence and mortality, which has brought a severe threat to human health during the past decades. Hantaviruses are transmitted to humans mainly via inhalation of virus-contaminated excreta and secreta, contaminated food, and rodent bites (1). Therefore, the outbreaks of HFRS is strongly relative with the rodent densities, and the vitality and infectivity of hantavirus, which depends on the changes of their natural living environment, such as temperature, precipitation, relative humidity, wind velocity, sunshine elevation, land use, normalized difference vegetation index (NDVI), temperature vegetation dryness index (TVDI) and El Niño-Southern Oscillation (ENSO) circulation (6, 18–22). Furthermore, urbanization and vaccination have impact on the frequency of contact between the human and rodent populations, and the human infection rates, which also contribute to the HFRS epidemic (23). All the above factors have potential regularity and periodicity and should be predictable. Forecasting the outbreaks of HFRS could give the hygiene authorities sufficient time to prepare for disseminating warnings and implementing public health interventions, such as exterminating rats, promotion of the vaccine in high-risk populations, as well as improving the living and working environment.

The GIS-based spatiotemporal analyses have been widely used to analyze the changing patterns of infectious diseases in the field of surveillance and infectious disease management (24, 25). Here, our GIS mapping showed significant changes in the spatiotemporal dynamics of HFRS throughout mainland China during 2004–2018. The HFRS outbreaks in northeast China (traditional high epidemic areas) has declined since 2005, mainly due to the implementation of effective vaccination, improvement of the environment, and rodent control measures in these areas (1).

Time-dimension analysis of the incidence of infectious disease could help to explain the observed epidemic status, subsequently establish a quality control system, and reallocate public health resources. As above mentioned, long-term changes in climate, environment, rodent abundance, and social features of human populations have generated non-stationarities in numerous epidemics. To overcome the problems of analyzing non-stationary time-series, wavelet analysis was applied to characterize them and estimate dependencies among non-stationary signals. Wavelet analysis is particularly attractive in view of epidemiological and environmental time-series and the relationships between them (9). The application of wavelet analysis has advantages in analyzing the underlying relevance in both time and frequency domain (26). WPS estimates the spectrum as a function of time and could reveal the time-series change of different periodic components over time (27), therefore, WPS was applied to study the outbreak cycles of HFRS in mainland China.

Traditional time-series models are designed to reveal the linear functional relationship between the current incidence and historical incidence of infectious diseases and establish linear regression models to predict their incidence in the future (28, 29). The application of ANN in epidemiological research has strength in self-learning, self-organization, good fault tolerance, and excellent non-linear approximation. As one kind of ANN, the WNN could be used in analyzing infectious diseases (30). In this study, we used the analytical approach of the wavelet to investigate HFRS outbreak cycles from January 2004 to December 2019 in mainland China, and the WNN to predict the HFRS incidence. Our results disclosed the periodicity of HFRS incidence in mainland China, two peaks per year in summer and winter, which could help to allocate resources to deal with HFRS outbreaks. These results showed that the WPS and WNN model performed well in analyzing historical periodicity and forecasting the incidence of HFRS outbreaks. These procedures can also be used in analyzing HFRS epidemic in other countries or regions, and other infectious diseases with outbreak period.

Our study has limitations. First, the factors of climate, environment, rodent density, and urbanization level, which might have important impacts on HFRS epidemic, were not included in this study due to data unavailable. Second, besides the WNN, there are other non-linear approaches that could predict the incidence of infectious diseases, such as the back-propagation neuron network (BPNN), and support vector machine (SVM). Therefore, the accuracy of WNN prediction was also compared with BPNN and SVM using parameters of MSE, RMSE, NRMSE, and MAPE (Supplementary Table 2). All parameters of WNN were the smallest, followed by SVM, and then BPNN, these results indicated that the prediction effect of WNN was the best for our dataset.

## Conclusions

This study explored the spatiotemporal features of HFRS from 2004 to 2019 in mainland China using GIS, the cyclical fluctuation of HFRS was described by the WPS, and a WNN model was constructed to monitor and predict the trends of HFRS outbreaks. Our results could provide valuable tools for the hygiene authorities to design and implement effective measures for the control and prevention of HFRS in China.

## Data Availability Statement

Publicly available datasets were analyzed in this study. This data can be found at: The datasets for this study can be found in the www.chinacdc.cn and the China Public Health Statistical Yearbook.

## Author Contributions

L-XZ and LS: conception or design of the work, acquisition, analysis, or interpretation of data for the work, and drafting the work or revising. All authors: contributed to the article and approved the submitted version.

## Conflict of Interest

The authors declare that the research was conducted in the absence of any commercial or financial relationships that could be construed as a potential conflict of interest.
