# Supplementary material for: Analysis of Hemorrhagic Fever With Renal Syndrome Using Wavelet Tools in Mainland China, 2004–2019
Source: Front Public Health. 2020 Dec 1;8:571984. doi: 10.3389/fpubh.2020.571984 (PMC7736046; doi:10.3389/fpubh.2020.571984)
Supplement: Supplementary file 1 [file Data_Sheet_1.docx]

Supplementary Material

# Supplementary Tables

Table S1. MSE, RMSE, NRMSE, and MAPE for different WNN models.

| WNN structure | 12-1-1 | 12-2-1 | 12-3-1 | 12-4-1 | 12-5-1 | 12-6-1 | 12-7-1 | 12-8-1 | 12-9-1 | 12-10-1 | 12-11-1 | 12-12-1 |
| --- | --- | --- | --- | --- | --- | --- | --- | --- | --- | --- | --- | --- |
| MSE | 0.0064 | 0.0064 | 0.00029 | 0.000347 | 0.000449 | 0.00019 | 0.0056 | 0.00034 | 0.000496 | 0.000247 | 0.000764 | 0.000394 |
| RMSE | 0.0801 | 0.08 | 0.017 | 0.0186 | 0.0212 | 0.0138 | 0.075 | 0.0184 | 0.0223 | 0.0157 | 0.0276 | 0.0198 |
| NRMSE | 59.5576 | 129270 | 0.1312 | 0.2003 | 0.2211 | 0.1064 | 0.9197 | 0.1543 | 0.1684 | 0.1234 | 0.187 | 0.1676 |
| MAPE | 53.2488 | 53.2134 | 16.9733 | 23.4086 | 32.9633 | 12.54 | 51.5504 | 23.8077 | 21.1674 | 17.5202 | 47.9264 | 27.1344 |

Note: e.g. 12-6-1, that is, the input layer had 12 nodes, which meant that the incidence of 12 months before the set time point, hidden layer had 6 nodes, and the output layer has 1 node.

Abbr: MSE, Mean Square Error; RMSE, Root Mean Squared Error; NRMSE, Normalized Root Mean Squared Error; MAPE, Mean Absolute Percentage Error.

Table S2. MSE, RMSE, NRMSE, and MAPE for WNN, SVM, and BPNN.

| Approaches | WNN | SVM | BPNN |
| --- | --- | --- | --- |
| MSE | 1.90E-04 | 1.98E-04 | 2.92E-04 |
| RMSE | 0.0138 | 0.0141 | 0.0171 |
| NRMSE | 0.1064 | 0.1047 | 0.1272 |
| MAPE | 12.54 | 12.9571 | 19.7062 |

Note: the WNN structure is 12-6-1, and, BPNN structure is 12-6-1.

Abbr: SVM, Support Vector Machine; BPNN, Back Propagation Neuron Network; MSE, Mean Square Error; RMSE, Root Mean Squared Error; NRMSE, Normalized Root Mean Squared Error; MAPE, Mean Absolute Percentage Error.
